# Is the detection of hepatocellular carcinoma in liver transplantation candidates impaired using a dedicated CMR stress test as a 'one-stop-shop'? a pathological correlation study

**DOI:** 10.1186/1532-429X-16-S1-P86

**Published:** 2014-01-16

**Authors:** Sahadev T Reddy, Ngoc L Thai, Mark Doyle, Jose Oliva, Kususm B Tom, Rishi Maheshwary, June A Yamrozik, Geetha Rayarao, Ronald B Williams, Moneal Shah, Jacqueline Poydence, Diane V Thompson, Robert W Biederman

**Affiliations:** 1 Cardiac MRI, Allegheny General Hospital, Pittsburgh, Pennsylvania, USA

## Background

Precise diagnosis and staging of hepatocellular carcinoma (HCC) is crucial in the selection and timing of orthotopic liver transplantation (OLT) patients, especially cirrhotics. The preoperative workup of OLT patients is logistically cumbersome and expensive given the need for separate cardiac, vascular, and abdominal imaging. We have recently demonstrated the value of a 'one-stop-shop' approach combining the attributes of a nuclear stress, echocardiogram and abdominal MRI into one single pre-op CMR evaluation. However, this approach requires further validation as accurate detection of HCC is critically important. Hypothesis: HCC detectability is independent of whether the study is performed as part of a larger one-stop-shop CMR or as a traditional focused MRI. Objective: We evaluated the 'detectability' of HCC using cardiac MRI for pre-operative evaluation of OLT candidates in a CMR suite performing assessment of cardiac structure, function and viability, along with simultaneous evaluation of thoracoabdominal vasculature and liver anatomy and compared with histopathology in explanted livers.

## Methods

In this pilot study, patients (n = 51) underwent a standard cardiac exam, stress CMR, and abdominal MRI on a dedicated CMR scanner during a single imaging session. Abdominal MRA along with liver parenchymal imaging was obtained via a Liver Acquisition with Volume Acquisition (LAVA) technique. Another representative cohort (n = 26) underwent standard abdominal MRI in a Radiology Suite. Pathological results at explanations were compared to interpretation of the CMR/MRI exams.

## Results

Over 3 years, 51/77 OLT candidates (56 ± 5 years, 35% F, MELD score of 11; range 6-40) underwent MRI in a dedicated CMR suit as a part of an integrated pre-operative evaluation of liver and heart. The remaining 26 pts underwent traditional pre-op evaluation of liver while cardiac stress testing was performed elsewhere via nuclear/echo. All referred pts completed standard dynamic CMR, 98% completed stress CMR, 94% completed liver MRI and 45 pts (88%) completing entire CMR exam. Nine pts (20%) in the combined CMR group and 12 (46%) in the traditional group proceeded to OLT. Of these, 2/9 pts in the CMR group and 2/12 pts in the traditional group were found to have lesions suggestive of HCC. These findings were all confirmed at explant surgical pathology with 100% detection/exclusion for HCC (100% NPV/PPV).

## Conclusions

In this Proof-of-Concept study, it appears feasible to perform a comprehensive pre-operative liver transplant evaluation in a CMR suite for the detection of HCC lesions. Our study confirmed that MRI evaluation of the liver along with cardiac preoperative evaluation is equivalent to traditional testing for the detection of HCC lesions, which was confirmed with 100% histopathological concordance. This is an important next step towards validation of the CMR One-Stop-Shop concept for pre-op evaluation for potential OLT recipients. The final step is a cost-effective analysis.

## Funding

Internal.

**Table 1:** 

Patient Characteristics	n = 51 (CMR Group)	n = 26 (Traditional Group)
Age (Mean ± SD)	56 ± 8	53 ± 10

Gender (Female %)	37%	37%

MELD score (Mean ± SD; Range)	11 ± 8; Range 6-40	22 ± 10; Range 6-40

Hepatic Encephalopathy (%)	39%	39%

Transplant Performed with stress test(%)	14% (9)	46% (12)

Transplant performed without stress test	0%	15% (4)

Transplant patients with HCC on liver MRI	2	2

HCC in explanted liver histopathology	2	2

Critically Ill	22%	10%

APACHE II Score	22 ± 7	23 ± 8

Etiology of Cirrhosis (%) 1. Cryptogenic/Unknown 2. Alcohol 3. NASH/NAFLD 4. Hepatitis C 5. Multiple Causes 6. Hepatitis B 7. Primary sclerosing cholangitis 8. Primary biliary cirrhosis 9. Cystic fibrosis 10. Hemochromatosis	31% 25% 17% 13% 6% 4% 4% 2% 2%	19% 12% 8% 22% 31% 4% 4% 2%

Patient demographics and characteristics, Traditional group under went pre-evaluation without cardiac MRI (HCC- hepatocellular carcinoma; MELD - model for end-stage liver disease).

